# Poisoning by Arsenic Successfully Treated with Calcined Magnesia

**Published:** 1848-10

**Authors:** 


*Poisoning by Arsenic successfully treated with Calcined Magnesia.* By Emory Bissel, M. D., of Norwalk, Conn.

Peter Galpin, a labourer, aged 27, a powerful and robust young man, of intemperate habits, attempted suicide on the evening of the 24th of March last, by taking arsenic. As is often the fact, he was prompted to the deed by those horrors and remorse of conscience which so often succeed a debauch. The quantity taken, as nearly as could be determined, was not far from a scruple. When I was first apprised of the fact, two hours had elapsed from the time it was taken. I hastened the messenger who came for me as quickly back as possible, with some thirty grains of sulphate of zinc—in two doses—with directions to administer it as soon as he could reach home, on horseback—distance about one mile—to give the second parcel in ten minutes, if needful. I followed as expeditiously as I could; when I arrived he had vomited freely twice, but without any relief. The family had given him copious draughts of a weak infusion of tobacco, which produced no other effect than to increase his sufferings, which at this time were extreme; so much so, that he begged me to kill him at once, if I could not end his pain in any other way. His pulse was one hundred and thirty per minute, small and wiry, He complained of great constriction and dryness of the fauces, but chiefly of a most agonizing pain and burning in the stomach; it seemed, as he expressed it, "as if it were filled with burning coals." As nearly three hours had now elapsed, since the poison entered the stomach, I considered that any further effort to evacuate it would be futile, and that if life was saved at all, it must be by the antidotal power of some medicinal agent Having had my attention directed to the experiments of Prof. Peter, of Transylvania University, and the case of Lepage, published in the January and April number of this Journal, in 1847, I determined to give the calcined magnesia a fair trial, and accordingly put it up in drachm doses, to be given every hour, mixed in milk and water. During the hour I remained with him, his symptoms were rapidly becoming more unfavorable. The pulse was one hundred and fifty per minute, the constriction and dryness of the fauces extreme, the whole surface bedewed with perspiration, the pain and burning sensation in the stomach seemed augmented to the highest possible degree, whilst the right hand was entirely paralyzed; in short, every thing betokened a speedy dissolution. I left him at 10 o'clock in charge of the mistress of the family, whom I knew to be an intelligent and faithful nurse. On visiting him the next morning, instead of finding him dead, as I much feared, I was very happily surprised to find him very quietly dozing in an easy chair. I learned from the lady, who had been unremitting in her care of him during the whole night, that in a very few minutes (not more than five or ten) after taking the first dose of magnesia, he said he felt much relieved, and before the time came for the second dose, he had fallen into a doze. She stated that each successive dose had produced the most surprising and marked mitigation of every symptom, and that long before morning he was entirely freed from suffering, and had, on the whole, passed a quiet and comfortable night. The bowels had moved freely and easily twice during the time. He complained of nothing save a general weakness, and a sort of faintness at the pit of the stomach. The right hand had recovered its power, and the pulse had fallen to eighty-five per minute. Directed to continue the magnesia, through the day, once in four hours, and to give light nourishment. On the sixth I made my third and last visit, as the young man seemed to require nothing but nursing, and a little time, for the recovery of his strength. I have since learned, that in a very few days he resumed his labor upon the farm, and felt no inconvenience from what he had taken, except a muscular weakness of the lower extremities, which was not very great. The case, in some of its aspects, much resembles that of Lepage, especially in the prompt and efficacious effects of the remedy, and the speedy recovery which followed. Should future cases yield results as gratifying as these two we may, indeed congratulate the profession in the possession of a sovreign and prompt antidote, always at hand and easily administered, to the dreadful poison by which such numbers of our race have found a terrific and agonizing death.—*American Journal of Medical Sciences.*

